# Can Human Handling Increase the Presence of Multidrug Resistance (MDR) in *Salmonella* spp. Isolated from Food Sources?

**DOI:** 10.3390/microorganisms9102018

**Published:** 2021-09-23

**Authors:** Valeria Gargano, Delia Gambino, Sergio Migliore, Maria Vitale, Sonia Sciortino, Antonella Costa, Domenico Vicari

**Affiliations:** Istituto Zooprofilattico Sperimentale della Sicilia, 90129 Palermo, Italy; valeria.gargano@izssicilia.it (V.G.); deliagamb@gmail.com (D.G.); maria.vitale@izssicilia.it (M.V.); sonia.sciortino77@gmail.com (S.S.); antonella.costa@izssicilia.it (A.C.); domenico.vicari@izssicilia.it (D.V.)

**Keywords:** MDR, multi-drugs resistance, *Salmonella*

## Abstract

The spread of antibiotic resistance (AR) among zoonotic pathogens is a serious health problem, especially because in the last decade the massive use of antibiotics has favored the emergence of Multidrug Resistance (MDR) strains. Some species of the *Salmonella* genus are among the major causes of foodborne infections worldwide and could represent reservoirs of AR. For these reasons, the susceptibility to six antibiotic classes of 63 strains isolated from animals and food was determined to assess the presence of MDR strains. In addition, the detection of resistance genes was done for strains that resulted in MDR. A statistically significant difference was found when comparing the presence of *Salmonella* spp. MDR strains between strains isolated from animals and strains isolated from food. Our data seem to indicate that MDR occurs mostly in *Salmonella* strains isolated from food.

## 1. Introduction

Antibiotic resistance (AR) occurs when a drug becomes ineffective against bacterial infections. Several of these bacteria can infect humans and animals. Antibiotic resistance leads to higher medical costs, longer hospital stays, and increased mortality. In the last decade, the occurrence of AR has increased so strongly that a public health impact assessment, specific to pathogen, antibiotic, and geographic area, has become necessary [1]. Since the middle of the last century, large amounts of antibiotics in human and veterinary medicine have been used, and this practice has greatly amplified the selection of antibiotic-resistant microorganisms. However, the problem of antibiotic resistance is complex because it is also due to the use of antibiotics in animal husbandry and agriculture, the spread of hospital infections caused by antibiotic-resistant microorganisms (and the limited control of these infections), and a greater spread of resistant strains due to increased international travel and migration flows [2]. In addition, the emergence of pathogens resistant simultaneously to three or more classes of antibiotics (MDR) further reduces the possibility of effective treatment [3]. Thus, international organizations including the World Health Organization (WHO), the European Union (EU), and the European Centre for Disease Prevention and Control (ECDC) have published guidelines and proposed coordinated strategies and actions to contain AR as a healthcare priority [1]. In fact, AR is one of the major public health problems worldwide with important implications both from a clinical point of view and in terms of economic impact. Additional costs are required for the use of more expensive drugs and procedures, longer hospital stays, and potential consequent severe disability. The World Health Organization (WHO) has stated that MDR infections could result in an estimated 2.4 million deaths in Europe, North America, and Australia between 2015 and 2050 if efforts to stem the spread of antibiotic resistance are not intensified [4]. However, in three out of four cases, these deaths could be prevented by implementing simple hygiene measures and more prudent antibiotic prescribing. Truly a short-term investment aimed at curbing the wide spread of resistant bacteria would save lives and save money in the long run.

In a 2014 World Economic Forum report, the 50 global risks were analyzed in terms of their economic, environmental, geopolitical, social, and technological consequences and ranked according to their likelihood of impact, which for AR was considered as high as terrorism or climate change. Recently, the potential use of multidrug-resistant agents as a biological weapon has become of particular concern [4].

For these reasons, actions to promote prudent use of antibiotics and prevent the spread of existing infections among humans should be combined with similar interventions in other areas in the context of a truly holistic (One Health) approach to health.

Salmonellosis is a disease of bacterial etiology that is widespread throughout the world and affects many animals, including humans. In farm animals, the infection is generally presented with clinical sporadic forms and rarely with real outbreaks. The manifested forms may be enteric with symptoms of varying severity such as diarrhea (from watery to bloody), dehydration, weakness, inappetence, and sometimes fever [5]. Abortigenic forms are possible, which can be of particular economic importance in sheep (*Salmonella* Abortus ovis) [6]. Similar symptoms can also occur in pets, while wildlife seem to be healthy reservoirs of this pathogen that can be disseminated in the environment through animal feces [7]. In humans, the disease is mainly food borne in origin and can have various forms of severity, with clinical conditions characterized by mild symptoms to life-threatening disease, especially when occurring in pediatric, elderly, and immunocompromised patients. Severe forms of salmonellosis require antibiotic therapy, in both human and veterinary medicine, with quinolones, beta lactamases, aminoglycosides, tetracyclines, and sulfamethoxazole-trimethoprim.

WHO has grouped pathogens into a list of priority, dividing them by species and type of AR into three priority levels: critical, high, and medium. Specifically, *Salmonella* spp. is present in priority group I, which establishes a critical level of attention for carbapenemase- and third-generation cephalosporin-resistant *Enterobacteriales*, and in priority group II, quinolone-resistant bacteria [8]. Furthermore, the overuse of antibiotics has also promoted in *Salmonella* spp. the emergence of MDR strains, which represent a significant health problem. In fact, it is estimated that of the 100,000 cases/year of salmonellosis a large proportion are due to MDR strains [1]. In addition, recently emerging strains of *Salmonella* isolated from humans have been found to be resistant to extended cephalosporins, quinolones, tetracyclines, ampicillin, and sulfonamides with cases of MDR, in which the origin could be traced back to poultry farms [9].

For these reasons, the present study aimed to investigate the antibiotic susceptibility profile of 63 strains of *Salmonella* spp. isolated from animals and food sources in order to assess the presence of MDR strains.

## 2. Materials and Methods

### 2.1. Collection of Salmonella spp. Strains

A total of 63 *Salmonella* spp. strains were isolated and analyzed in this study. Of these, 47 originated from animal samples (group p1) that arrived in our laboratories for diagnostic purposes. In particular, 10 strains were isolated from ruminant feces samples (9 sheep and 1 bovine); 7 strains came from slaughterhouse sampling of pig carcasses; 12 strains came from dog feces; the remaining samples came from wild animals housed in a wildlife rescue center and in a zoo or found dead in wildlife. Finally, 16 strains were isolated from foodstuffs (group p2), that were subjected to food controls according to the European Community Legislation. The isolated strains and their source are reported in Table S1.

### 2.2. Antibiotic Susceptibility Assay Using the Disc Diffusion Method

The antibiotic susceptibility profile of 63 *Salmonella* spp. strains using the disc diffusion method (Kirby-Bauer) was investigated. Antibiograms were performed on Muller Hinton agar (Oxoid, Basingstoke, UK) according to Clinical and Laboratory Standard Institute (CLSI) guidelines [10]. Fifteen most commonly used antibiotics were selected: kanamycin (KAN, 30 µg), gentamicin (GEN, 10 µg), tobramycin (TOB, 10 µg), ampicillin (AMP, 10 µg), amoxicillin + clavulanic acid (AMC, 30 µg), cefotaxime (FOT, 30 µg), ceftriaxone (CRO, 30 µg), imipenem (IMI, 10 µg), chloramphenicol (CHL, 30 µg), nalidixic acid (NAL, 30 µg), enrofloxacin (ENR, 5 µg), ciprofloxacin (CIP, 5 µg), levofloxacin (LEV, 5 µg), sulfamethoxazole + trimethoprim (SXT, 23.75 + 1.25 µg), tetracycline (TET, 30 µg).

The strains were classified as resistant (R), intermediate (I), or susceptible (S) according to the CLSI ranges [10].

### 2.3. MIC Determination for MDR Strains

Minimum Inhibitory Concentration (MIC) values (µg/mL) were determined using the 96-well Sensititre™ EU Surveillance *Salmonella*/*E. coli* EUVSEC plates (Thermo Scientific, Waltham, MA, USA), according to the manufacturer’s instructions. These plates contained scalar dilutions (2-fold dilutions) for the following 14 antibiotics: sulfamethoxazole (SMX, 8-1024), trimethoprim (TMP, 0.25-32), ciprofloxacin (CIP, 0.015-8), tetracycline (TET, 2-64), meropenem (MERO, 0.03-16), azithromycin (AZI, 2-64), nalidixic acid (NAL, 4-128), cefotaxime (FOT, 0.25-4), chloramphenicol (CHL, 8-128), tigecycline (TGC, 0.25-8), ceftazidime (TAZ, 0.5-8), colistin (COL, 1-16), ampicillin (AMP, 1-64), and gentamicin (GEN, 0.5-32).

Results obtained from manual plate reading using Sensititre™ Manual Viewbox (Thermo Fisher Scientific, Waltham, MA, USA) were interpreted in accordance with CLSI breakpoints [10].

### 2.4. Detection of Antibiotic Resistance Genes in MDR Strains

The presence of resistance genes to beta-lactams, tetracyclines, and sulphonamides was determined by PCR using the primers shown in Table 1 and following previously published protocols [11]. Subsequently, the amplicons were used for electrophoresis on a 2% agarose gel to determine the product size. To confirm the presence of antibiotic resistance genes, at least one gene from each type sought was sequenced according to the method previously described and used as a positive control [11].

### 2.5. Statistical Analysis

In order to evaluate statistically the presence of MDR in the different isolated strains, the analysis was performed using chi-square test calculator on the Social Science Statistics website [14]. Proportion differences between the strains isolated from animals (group p1) and food (group p2) sources were tested. Statistical analysis within the p2 group was also calculated to evaluate the proportion differences of MDR between poultry and bovine meats. A value of *p* < 0.05 was considered significant. The null hypothesis asserts no differences among groups.

## 3. Results

### 3.1. Antibiotic Susceptibility Assay Using the Disc Diffusion Method

Antibiotic susceptibility testing showed that none of the 63 *Salmonella* spp. strains isolated in the samples were resistant to amoxicillin/clavulanic acid, ceftriaxone, imipenem, ciprofloxacin, enrofloxacin, levofloxacin, and chloramphenicol. In addition, only 3/63 strains (4.7%) isolated from food sources were resistant to cefotaxime, while the only strain resistant to both gentamicin and tobramycin was isolated from a dog. In contrast, resistance to kanamycin, (6/63, 9.5%), sulfamethoxazole/trimethoprim (7/63, 11.1%), and nalidixic acid (8/63, 12.6%) was found prevalently in food sources. Moreover, high resistance to ampicillin (14/63, 22.2%) and tetracycline (21/63, 33.3%) was detected both in animal than in food sources, with 19.1% and 39.5% for ampicillin and tetracycline, respectively, in animal samples and 31.2% and 56.2% for ampicillin and tetracycline, respectively, in food sources (Table S1).

The disc diffusion tests revealed nine MDR *Salmonella* spp. strains, isolated prevalently from food sources: one *S.* Typhimurium (ID.17 from a dog), seven *S.* Infantis (ID.50 and ID.51 from Bovine meat, ID. 52, ID.53, ID.57, and ID.58 from poultry meat, and ID.36 from *Carduelis carduelis*), and one *S.* Newport (ID.49 from Turkey meat). Specifically, two strains showed resistance to three antibiotic classes: strain ID.17 to aminoglycosides, beta-lactams, and tetracyclines, and strain ID.57 to quinolones, sulfonamides, and tetracyclines. Five strains to four classes: strain ID.49 to aminoglycosides, beta-lactams, sulfonamides, tetracyclines; ID.50 to aminoglycosides, beta-lactams, quinolones, and tetracyclines; strain ID.52 to beta lactams, quinolones, sulfonamides, and tetracyclines; strains ID.53 and ID.58 to aminoglycosides, quinolones, sulfonamides, and tetracyclines. Finally, strains ID.36 and ID.51 showed resistance to five classes: aminoglycosides, beta-lactams, quinolones, sulfonamides, and tetracyclines.

### 3.2. MIC Determination

Determination of the minimum inhibitory concentration (MIC) for the nine strains that resulted in MDR was performed. However, multi-drug resistance was confirmed for only eight strains, as strain ID. 17 did not provide MIC values (µg/mL) that indicated resistance to gentamicin. The other eight strains were all resistant to sulfonamides and tetracyclines classes. In addition, with the exception of strain ID.49, all strains were resistant to quinolones; strains ID. 50, 51 and 52 were also resistant to beta-lactams, and strains ID. 49 to aminoglycosides (Table 2).

### 3.3. Antibiotic Resistance Gene Detection

**Figure 1:**
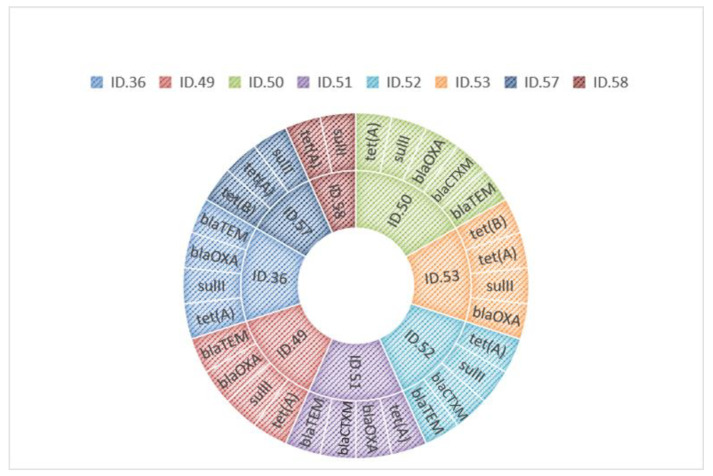
ARGs harbored by each MDR strain.

Molecular investigations revealed in these strains the presence of *bla*_TEM_, *bla*_CTXM_, *bla*_OXA_, *sul*II, *tet*(A), and *tet*(B) genes. Specifically, all strains harbored the *tet*(A) gene, seven strains harbored the *sul*II gene, five harbored the *bla*_OXA_ gene, whereas *bla*_TEM_ and *bla*_CTXM_ were present in three strains. Instead, no strain was found to harbor the *bla*_SHV_, *sul*I, and *sul*III genes. However, when observing the MDR strains data, it is found that 7/8 (87.5%) of these *Salmonella* spp. were from food samples and only 1/8 (12.5%) were from animal samples. Table 3 shows the comparison of detected phenotypic resistances and the presence of resistance genes in the eight MDR strains. In Figure 1, ARGs harbored by each strain were reported. 

### 3.4. Statistical Analyses

The compared proportions of MDR stains originating from animal (p1 = 1/47) and food (p2 = 7/16) sources showed a statistically significant difference with a *p*-value < 0.001 at 95% confidence level. In contrast, no statistical differences within p2 group were found compared the difference of proportion of MDR between poultry and bovine meats.

## 4. Discussion

In this study, we showed the results on MDR analysis in 63 *Salmonella* spp. strains. In particular, we investigated the antibiotic susceptibility profile and the presence of the antibiotic resistance genes (ARGs) in 47 and 16 strains isolated from animal food sources respectively. Susceptibility testing conducted with the two methods resulted in the detection of 8/63 MDR strains. In fact, although 9/63 MDR strains were detected with the Kirby-Bauer method, the quantitative assay (MIC) did not confirm the gentamicin resistance for the *S. enterica* subsp. Typhimurium strain from dog (ID. 17), and only 8/9 strains confirmed their MDR profile.

Phenotypic resistances for each MDR strain were confirmed by detection of related resistance genes. The most represented gene was *tet*(A) in all strains (8/8), followed by *sul*II (7/8), and *bla*_TEM_ (5/8). In particular, *bla*_OXA_ and *bla*_TEM_ genes were found in all ampicillin-resistant strains, *bla*_CTXM_ in 3rd generation cephalosporin-resistant strains. Regarding tetracycline resistance, all strains had at least one *tet* resistance gene and 2/8 strains had both *tet*(A) and *tet*(B); furthermore, all sulfonamide-resistant strains harbored the *sul*II gene. This aspect is particularly interesting because antibiotic resistance genes (ARGs) are often harbored together with genetic elements that make them extremely mobile (transposomes, introns, plasmids, etc.). This feature makes them easily transferable to other bacteria that can thus acquire antibiotic resistance. The presence of these ARGs in foodborne bacteria, such as *Salmonella* spp., can transfer these genes to the intestinal bacterial flora through its great genomic plasticity, contributing significantly to the spreading of antibiotic resistance.

Our data seem to suggest that MDR strains are primarily represented by Infantis serovars, which EFSA lists as one of the five most prevalent serovars in human infections in the EU along with enteritidis, typhimurium, and its monophasic variant, and newport [15].

Regarding *Salmonella* spp. isolated from wildlife and breeding animals, no MDR strains were found, and this is in accordance with other studies carried out in Sicily, which have proved a low presence of AR in bacterial strains isolated from these kinds of animals [7,16]. The selective pressure due to antibiotic use may be low under these conditions as Sicilian breeders often do not invest time and money in animal care and prefer to use vaccination and the isolation of sick animals from the production process [17].

MDR strains represented the 12.6% (8/63) of our *Salmonella* spp. isolated: one strain came from a goldfinch, while the other seven came from food.

The resistance found in the strain isolated from a goldfinch hosted in a zoo could be due to the close proximity with humans: as suggested by other authors, humans could represent a primary reservoir of AR bacterial strains and could transfer these genetically-based resistances to pets [18].

A significant difference was found between the presence of MDR *Salmonella* spp. strain in the two compared groups. Indeed, the presence of *Salmonella* MDR was detected in 7/16 strains isolated from food and, in particular, in ready-to-cook meat preparation. All these foodborne strains (7/7) were resistant to the tetracycline class, 6/7 to the sulfonamide class, and 4/7 to the beta-lactams class. Moreover, the resistance to tigecycline in all MDR strains and to cefotaxime in 3/8 MDR strains is an interesting fact, because these two antibiotics are used against nosocomial infections, [19].

Analyzing the overall data, it can be observed that the presence of *Salmonella* MDR strains is greater as the proximity of the sample to human manipulation increases. The statistically significant difference between the two groups analyzed (strains from animals and strains from food) would contribute to strengthen this hypothesis also since meat preparations require a lot of handling. Although salmonellosis is one of the main foodborne disease [15], the spread of AR in food-borne strains could be related to humans; since the inappropriate and arbitrary use of antibiotics is still widespread in human medicine and *Salmonella* spp. are optimal reservoir of AMR, as demonstrated by other authors [20,21].

The emergence, diffusion, and persistence of AR remains a huge global health issue. Intensive animal husbandry, in particular, poultry, is considered a substantial portion of the global antibiotic use [22]. Indeed, 5 out of 7 *Salmonella* spp. strains originated from poultry meat, and no statistical difference was detected with respect to MDR strains from bovine meat. 

## 5. Conclusions

The spread of antibiotic-resistant strains is a serious health problem that affects animals and humans. Although in recent decades, actions to reduce the use of antibiotics have been undertaken, AR is still considered a zoonosis and represents a serious health threat. In this regard, an important issue concerns the prevalence of MDR in bacteria such as *Salmonella* spp. responsible for globally widespread foodborne diseases. Indeed, MDR makes antibiotic treatment of infection difficult and leads to higher medical costs, prolonged hospital stays, and higher mortality. This study confirms the ubiquitous presence of *Salmonella* spp. in several sources, bringing out the difference in MDR strains between animal and food sources.

Our preliminary data speculate that the presence of MDR strains increases in samples subject to human manipulation. Consequently, understanding which animals and humans are the real sources of resistant bacterial strains could be crucial to limit the occurrence of MDR bacteria and better protect public health.

## Figures and Tables

**Table 1:** Primers used in this study.

Target Name	Resistance Mechanism	Amplicon Size (bp)	Reference
*bla*_TEM_	β-Lactamases	858	[12]
*bla*_CTXM_	β-Lactamases	112	[7]
*bla*_SHV_	β-Lactamases	807	[7]
*bla*_OXA_	β-Lactamases	590	[7]
*tet*(A)	Efflux	210	[7]
*tet*(B)	Efflux	659	[13]
*sul*I	Dihydropteroate synthase inhibitor	316	[13]
*sul*II	Dihydropteroate synthase inhibitor	191	[7]
*sul*III	Dihydropteroate synthase inhibitor	799	[13]

**Table 2:** MIC values (µg/mL) for MDR strains (*n* = 9).

Antibiotics Classes	Antibiotics	Strains								
		ID. 17	ID. 36	ID. 49	ID. 50	ID. 51	ID. 52	ID. 53	ID. 57	ID. 58
Aminoglycosides	GEN	≥8	≤0.5	≤0.5	≥1	≤0.5	≤0.5	≥1	≤0.5	≤0.5
Beta lactams	AMP	**≥64**	**>64**	**>64**	**>64**	**>64**	**>64**	≥4	≤1	≥2
	TAZ	≥0.5	≤0.5	≤0.5	≥4	≥4	≥4	≤0.5	≤0.5	≥1
	FOT	≤0.25	≤0.25	≤0.25	**>4**	**>4**	**>4**	≤0.25	≤0.25	≥0.5
	MERO	≤0.03	≤0.03	≤0.03	≤0.03	≤0.03	≤0.03	≤0.03	≤0.03	≤0.03
Phenicoles	CHL	≤8	≥16	≤8	≤8	≤8	≥16	≤8	≤8	≤8
Polymixine	COL	≤1	≤1	≤1	≤1	≤1	≤1	≥4	≤1	≤1
Quinolones	NAL	≥8	**>128**	≥16	**>128**	**>128**	**>128**	**>128**	**>128**	**>128**
	CIP	≥0.03	≥0.25	≥0.5	≥0.12	≥0.25	≥0.25	≥0.5	**≥1**	≥0.25
Sulfonamides	SMX	≥64	**>1024**	**>1024**	**>1024**	**>1024**	**>1024**	**>1024**	**>1024**	**>1024**
	TMP	≤0.25	**>32**	**>32**	**>32**	**>32**	**>32**	**>32**	**>32**	**>32**
Tetracyclines	TET	**>64**	**>64**	**>64**	**≥64**	**>64**	**>64**	**>64**	**>64**	**>64**
	TGC	**≥1**	**≥2**	**≥2**	**≥1**	**≥1**	**≥2**	**≥1**	**≥2**	**≥1**
Macrolides	AZI	≥8	≥8	≥8	≥16	≥4	≥4	≥16	≥8	≥8

Gentamicin (GEN, 0.5–32 µg/mL); ampicillin (AMP, 1–64 µg/mL); ceftazidime (TAZ, 0.5–8 µg/mL); cefotaxime (FOT, 0.25–4 µg/mL); meropenem (MERO, 0.03–16 µg/mL); chloramphenicol (CHL, 8–128 µg/mL); colistin (COL, 1–16 µg/mL); nalidixic acid (NAL, 4–128 µg/mL); ciprofloxacin (CIP, 0.015–8 µg/mL); sulfamethoxazole (SMX, 8–1024 µg/mL); trimethoprim (TMP, 0. 25–32 µg/mL); tetracycline (TET, 2–64 µg/mL); tigecycline (TGC, 0.25–8 µg/mL); azithromycin (AZI, 2–64 µg/mL); resistances are represented in bold.

**Table 3:** Genotypic and phenotypic resistance results.

Strains	Source	Serotype	Phenotypic Resistance	Resistance Genes
*ID. 36*	*Carduelis carduelis*	*S. enterica* subsp. Infantis	AMP, SMX, TET, TGC	*bla*_TEM_*, bla*_OXA_*, sul*II*, tet*(A)
*ID. 49*	Turkey meat	*S. enterica* subsp. Newport	AMP, SMX, TET, TGC	*bla*_TEM_*, bla*_OXA_*, sul*II*, tet*(A)
*ID. 50*	Bovine meat	*S. enterica* subsp. Infantis	AMP, FOT, SMX, TET, TGC	*bla*_TEM,_*bla*_CTXM_*, bla*_OXA_*, sul*II*, tet*(A)
*ID. 51*	Bovine meat	*S. enterica* subsp. Infantis	AMP, FOT, TET, TGC	*bla*_TEM,_*bla*_CTXM_*, bla*_OXA_*, tet*(A)
*ID. 52*	Poultry meat	*S. enterica* subsp. Infantis	AMP, FOT, SMX, TET, TGC	*bla*_TEM_*_,_bla*_CTXM_*, sul*II*, tet*(A)
*ID. 53*	Poultry meat	*S. enterica* subsp. Infantis	SMX, TET, TGC	*bla*_OXA_*, sul*II*, tet*(A)*, tet*(B)
*ID. 57*	Poultry meat	*S. enterica* subsp. Infantis	SMX, TET, TGC	*sul*II*, tet*(A)*, tet*(B)
*ID. 58*	Poultry meat	*S. enterica* subsp. Infantis	SMX, TET, TGC	*sul*II*, tet*(A)

Ampicillin (AMP); cefotaxime (FOT); sulfamethoxazole (SMX); tetracycline (TET); tigecycline (TGC).

## Data Availability

All data discussed are contained in the manuscript and Supplementary Materials.

## Supplementary Materials

The following are available online at https://www.mdpi.com/article/10.3390/microorganisms9102018/s1, Table S1: Source, serotyping and antibiotic resistance of *Salmonella* spp. analyzed (*n* = 63).
