# Supplementary material for: Can Human Handling Increase the Presence of Multidrug Resistance (MDR) in Salmonella spp. Isolated from Food Sources?
Source: Microorganisms. 2021 Sep 23;9(10):2018. doi: 10.3390/microorganisms9102018 (PMC8539229; doi:10.3390/microorganisms9102018)
Supplement: Supplementary file 1 [file microorganisms-09-02018-s001.zip › microorganisms-1393511-supplementary.pdf]

Table S1. Source, serotyping and antibiotic resistance of *Salmonella* spp. analysed (*n* = 63).

Id. Strain	Source	Serotyping	Antibiotic Resistance
1	Ovine	*S.* Typhimurium Monophasic Variant	AMP
2	Ovine	*S.* Typhimurium Monophasic Variant	AMP
3	Ovine	*S.* Montevideo	*
4	Ovine	*S.* Derby	*
5	Ovine	*S.* Elomrane	*
6	Ovine	*S.* Muenster	*
7	Ovine	*S.* Muenster	*
8	Ovine	*S.* Derby	*
9	Bovine	*S.* Muenster	*
10	Swine	*S.* Derby	*
11	Swine	*S.* Derby	*
12	Swine	*S.* Typhimurium	*
13	Swine	*S.* Derby	*
14	Swine	*S.* Derby	*
15	Swine	*S.* Typhimurium Monophasic Variant	TET
16	Swine	*S.* Kedougou	*
17	Dog	*S.* Typhimurium Monophasic Variant	GEN, TOB, AMP, TET
18	Dog	*S.* Goldcoast	TET
19	Dog	*S.* Goldcoast	TET
20	Dog	*S.* Typhimurium	AMP, TET
21	Dog	*S.* Typhimurium	AMP, TET
22	Dog	*S.* Typhimurium	*
23	Dog	*S.* Typhimurium	*
24	Dog	*S.* Enteritidis	*
25	Dog	*S.* Typhimurium	TET
26	Dog	*S.* Enteritidis	*
27	Dog	*S.* Elomrane	*
28	Dog	*S.* Schleissheim	*
29	*Python regius*	*S.* Bahrenfeld	*
30	*Trachemys scripta*	*S.* Richmond	*
31	*Iguana iguana*	*S.* Potsdam	*
32	*Trachemys scripta*	*S.* Kiambu	*
33	*Trachemys scripta*	*S.* Typhimurium	AMP, TET
34	*Carduelis carduelis*	*S.* Typhimurium	AMP, TET
35	*Nymphicus hollandicus*	*S.* Richmond	*
36	*Carduelis carduelis*	*S.* Infantis	KAN, AMP, NAL, SXT, TET
37	*Testudo hermanni*	*S.* Newport	TET
38	*Testudo hermanni*	*S.* Heron	*
39	*Testudo hermanni*	*S. bongori*	*
40	*Sus scrofa*	*S.* Elomrane	*
41	*Oryctolagus cuniculus*	*S.* Veneziana	*
42	*Testudo hermanni*	*S.* Abony	*
43	*Testudo hermanni*	*S.* Tennessee	NAL
44	*Ardea cinerea*	*S.* Enteritidis	*
45	*Stenella coeruleoalba*	*S.* Typhimurium	AMP, TET
46	*Caretta caretta*	*S.* Enteritidis	*
47	*Mobula mobular*	*S.* Typhimurium	*
48	Turkey meat	*S.* Typhimurium	*
49	Turkey meat	*S.* Newport	KAN, AMP, SXT, TET
50	Bovine meat	*S.* Infantis	KAN, AMP, FOT, NAL, TET
51	Bovine meat	*S.* Infantis	KAN, AMP, FOT, NAL, SXT, TET
52	Poultry meat	*S.* Infantis	AMP, FOT, NAL, SXT, TET

53	Poultry meat	*S.* Infantis	KAN, NA, SXT, TET
54	Poultry meat	*S.* Infantis	TET
55	Poultry meat	*S.* Kentucky	*
56	Poultry meat	*S.* Kentucky y	*
57	Poultry meat	*S.* Infantis	NAL, SXT, TET
58	Poultry meat	S. Infantis	KAN, NAL, SXT, TET
59	Eggs	S. Enteritidis	*
60	Mytiles	S. Aberdeen	AMP, TET
61	Mytiles	S. Bredeney	*
62	Mytiles	S. Bredeney	*
63	Clams	S. Kentucky	*

Kanamycin (KAN); Gentamicin (GEN); Tobramycin (TOB); Ampicillin (AMP); Cefotaxime (FOT); Nalidixic Acid (NAL); Sulphamethoxazole/Trimethoprim (STX); Tetracycline (TET); *no resistance detected.
